# Exosomal miRNAs in Lung Diseases: From Biologic Function to Therapeutic Targets

**DOI:** 10.3390/jcm8091345

**Published:** 2019-08-29

**Authors:** Julien Guiot, Ingrid Struman, Edouard Louis, Renaud Louis, Michel Malaise, Makon-Sébastien Njock

**Affiliations:** 1 Department of Respiratory Diseases, GIGA-I3 Research Group, University and CHU of Liège, 4000 Liège, Belgium; 2 Fibropôle Research Group, University and CHU of Liège, 4000 Liège, Belgium; 3 Laboratory of Molecular Angiogenesis, GIGA-Cancer Research Group, University of Liège, 4000 Liège, Belgium; 4 Department of Gastroenterology, GIGA-I3 Research Group, University and CHU of Liège, 4000 Liège, Belgium; 5 Department of Rheumatology, GIGA-I3 Research Group, University and CHU of Liège, 4000 Liège, Belgium

**Keywords:** exosomes, microRNAs, lung diseases, chronic obstructive pulmonary disease, asthma, acute lung injury/acute respiratory distress syndrome, idiopathic pulmonary fibrosis, diagnostic biomarkers, therapeutics

## Abstract

Increasing evidence suggests the potential role of extracellular vesicles (EVs) in many lung diseases. According to their subcellular origin, secretion mechanism, and size, EVs are currently classified into three subpopulations: exosomes, microvesicles, and apoptotic bodies. Exosomes are released in most biofluids, including airway fluids, and play a key role in intercellular communication via the delivery of their cargo (e.g., microRNAs (miRNAs)) to target cell. In a physiological context, lung exosomes present protective effects against stress signals which allow them to participate in the maintenance of lung homeostasis. The presence of air pollution alters the composition of lung exosomes (dysregulation of exosomal miRNAs) and their homeostatic property. Indeed, besides their potential as diagnostic biomarkers for lung diseases, lung exosomes are functional units capable of dysregulating numerous pathophysiological processes (including inflammation or fibrosis), resulting in the promotion of lung disease progression. Here, we review recent studies on the known and potential role of lung exosomes/exosomal miRNAs, in the maintaining of lung homeostasis on one hand, and in promoting lung disease progression on the other. We will also discuss using exosomes as prognostic/diagnostic biomarkers as well as therapeutic tools for lung diseases.

## 1. Introduction

Lung diseases are among the leading causes of death worldwide. Recently, the forum of international respiratory societies (FIRS) has reported the global burden of lung diseases [1]. Indeed, chronic obstructive pulmonary disease (COPD) affects 65 million people in the world, and about 3 million people die from it each year, making it the third leading cause of death worldwide [2,3]. More than 300 million people suffer from asthma [4], and about 4 million people annually die from acute lower respiratory tract infection [5]. Most lung diseases are due to smoke exposure and the poor quality of the air. As reported by FIRS, at least 2 billion people are exposed to household air pollution (toxic smoke of biomass fuel) [1] and 1 billion inhale polluted outdoor air. In addition, 1 billion people are exposed to tobacco smoke. The inhalation of smoke and unhealthy air (air pollution, allergens, and microbial pathogens) induces airway injury, which is a major risk factor in the development of lung diseases.

In a physiological context, airway epithelium is the first line of defense against environmental toxin, acting as a physical barrier to prevent the intrusion of pollutant agents or pathogens in the lung. Furthermore, airway epithelium contributes to lung homeostasis by regulating immune reactions and controlling tissue remodeling needed for repair after injury [6]. Repeated exposure of lung epithelium to gaseous pollution induces chronic airway inflammation and aberrant repair processes of lung, resulting in the development of lung diseases [7,8], such as interstitial lung diseases, COPD, or asthma.

In the past decades, extracellular vesicles (EVs) have emerged as essential actors of intercellular communication, particularly between epithelial cells and lung microenvironment. In this review, we aim to summarize current knowledge about the role of EVs, principally exosomes and exosomal microRNAs (miRNAs), in lung physiology and disease pathogenesis, including COPD, asthma, acute lung injury/acute respiratory distress syndrome (ALI/ARDS), and idiopathic pulmonary fibrosis (IPF). We also discuss the clinical development of exosome-based diagnosis and therapies for lung diseases.

## 2. Exosomes as Essential Actors of Intercellular Communication

### 2.1. Extracellular Vesicle Classification and Biogenesis

EVs are a heterogeneous population of small vesicles (30 nm to 5 µm) containing a phospholipid bilayer secreted from most, if not all, cell types [9]. These vesicles contain numerous bioactive molecules (nucleic acids, proteins, and lipids) which confer their biological activities. Cells can release three main types of EVs classified according to their sub-cellular origin, secretion mechanism, and size (Table 1): exosomes (30–150 nm), which are internal vesicles generated within late endosomes/multivesicular bodies (MVBs) and released to an extracellular environment after fusion of MVBs with the plasma membrane [10,11,12]; microvesicles (MVs) (also known as microparticles) are larger in size, 100 nm to 1 µm, and are formed by the outward budding and scission of plasma membrane [13]; and apoptotic bodies (ABs), the largest subtype of EVs (1–5 μm), which are released from plasma membrane of apoptotic cells [14,15].

The difficulty of isolating pure populations of each type of EVs should be noted, because their size and density ranges overlap. Indeed, most exosome isolation protocols do not isolate a pure population of exosomes, but a mixed population of small EVs. So, it is important to accurately characterize the isolated population for each study. A major ongoing challenge is to establish methods that will allow a good isolation of exosomes on one hand, and MVs on the other.

### 2.2. Exosome Composition and Function

Exosomes were first visualized in reticulocytes of rats [18] and sheep [19] in the mid-1980s. Initially, these vesicles were described as cellular waste and received little attention. Later, several studies highlighted the role of exosomes in physiological and pathological contexts, such as tumorigenicity, immune modulation, cardiovascular diseases, or lung diseases. In 2007, Valadi et al. were the first to demonstrate that mRNAs and miRNAs can be secreted from either human or mice mast cells encapsulated into exosomes and transferred to other mast cells [20]. Following this study, hundreds of others have reported the modulation of various signaling pathways involved in the development of lung diseases, including inflammatory and fibrotic pathways, via the transfer of select cargoes from a donor to a recipient cell. Exosomal cargoes are composed of genetic material (DNA, mRNA, non-coding RNAs including miRNAs and long non-coding RNA), proteins, and lipids. Exosomes are highly enriched in tetraspanins, including CD63, CD81, and CD9, which have been widely used as exosomal markers [21]. In addition, these vesicles present a high number of endosomal markers or markers of the endosomal sorting complex required for transport (such as tumor susceptibility gene 101 or ALG-2-interacting protein X), which reflects their endosomal origin [22].

Exosomes are constitutively secreted into biological fluids (e.g., bronchoalveolar lavage fluid (BALF), saliva, sputum, plasma) [23,24,25,26,27] and participate in intercellular communication via the transfer of their content (e.g., miRNAs) to target cells [28,29,30,31,32]. In a physiological context, exosomes play an important role in maintaining lung homeostasis. In the presence of airway injury, pulmonary cells release exosomes enriched with pro-inflammatory and pro-fibrotic-miRNAs which participate in the progression of lung diseases. In that context, interest in exosomes ranges from their function in the body to more translational applications, such as their use in diagnostics or therapeutics.

## 3. Exosomal microRNAs Play a Key Role in Lung Homeostasis

Intercellular communication between bronchial epithelial cells (ECs) and the broad range of cells that are present in the lung microenvironment is essential to keep the lung functioning properly. Bronchial EC-derived exosomes are major actors in the maintain of lung homeostasis through the regulation of inflammation and fibrotic processes. Indeed, Kesimer et al. have shown that epithelial-derived exosomes participate in innate mucosal defense [33]. These vesicles contain surface associated mucin (MUC)-1, MUC-4, and MUC-16, which confer them a neutralizing effect on the human influenza virus. Furthermore, Gupta et al. have shown that the transfer of exosomal cargo between airway ECs significantly alters the qualitative and quantitative profiles of airway secretions, including MUC hypersecretion, and the miRNA cargo of exosomes in target cells (including miR-34a/b/c, miR-449b/c, and miR-223) [34]. This finding indicates that epithelial-derived exosomes may be able to play an important role in airway biology and epithelial remodeling.

Recent studies highlighted the role of alveolar macrophage-derived exosomes on the modulation of inflammatory state within the lung. In an elegant study, Bourdonnay et al. have demonstrated that alveolar macrophage-derived exosomes are able to modulate inflammatory signaling by the transfer of suppressor of cytokine signaling (SOCS) 1 and 3 in alveolar epithelial cells and the inhibition of signal transducer and activator of transcription (STAT) activation [35]. The secretion of SOCS-enriched exosomes into the lung fluid was reduced by cigarette smoke (CS) exposure, diminishing their protective effect against lung inflammation [35]. An additional study by Ismail et al. revealed that MVs derived from alveolar macrophages are able to regulate airway inflammation through the transfer of miR-223 to various respiratory cells, including lung epithelial cells and monocytes [36].

Endothelial-derived EVs may also play a benefic role in lung homeostasis. Indeed, Njock el al. have demonstrated that endothelial-derived EVs are able to suppress monocyte activation by the delivery of anti-inflammatory miR-10a [28]. The immunomodulatory properties of these vesicles may protect the lung against inflammation that is responsible of several pulmonary diseases, such as COPD or ARDS.

**Figure 1:**
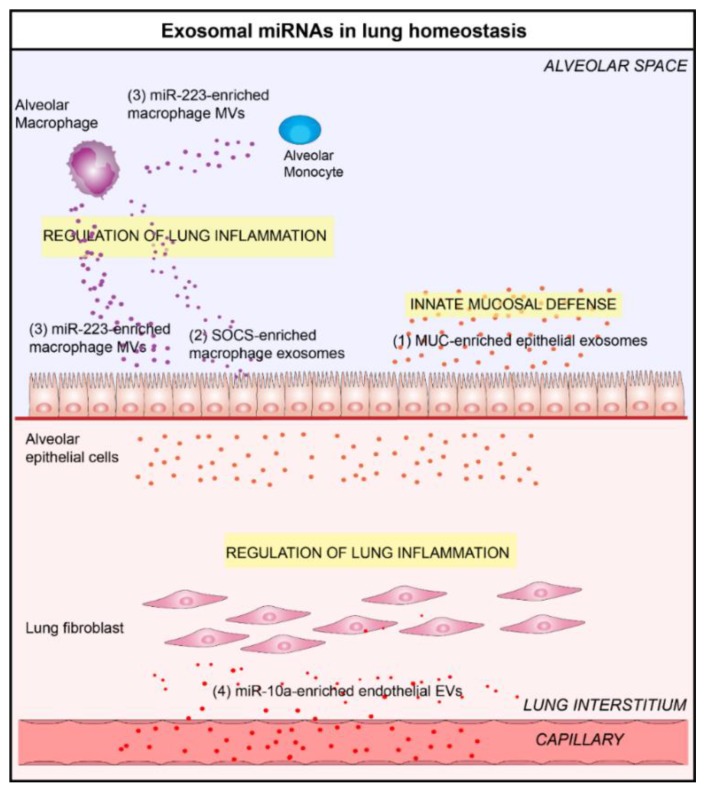
Lung extracellular vesicles (EVs) participate in the maintenance of lung homeostasis. (**1**) In a physiological context, bronchial epithelial cells (EC)-derived exosomes contain mucin (MUC) protein, which give them a neutralizing effect on human influenza virus and participate in innate mucosal defense [33,34]. (**2**) In the alveolar space, alveolar macrophage-derived exosomes are able to modulate inflammatory signaling by the transfer of suppressor of cytokine signaling (SOCS) proteins [35] or (**3**) miR-223 in various respiratory cells, including alveolar ECs and monocytes [36]. (**4**) Endothelial-derived EVs could also participate in lung homeostasis by delivering anti-inflammatory miR-10a to respiratory cells [28].

Based on the above findings, various cell types from the lung microenvironment participate in regulation of lung homeostasis via the transfer of anti-inflammatory/anti-fibrotic molecules (including miRNAs) to target cells (Figure 1).

## 4. Impact of Exosomal microRNAs in Lung Diseases

### 4.1. Chronic Obstructive Pulmonary Disease and Exosomal microRNAs

Inflammatory airway diseases are airway disorders that are related to the persistent inflammatory state of the lung, induced by noxious stimuli exposure (e.g., CS, allergens, infections, air pollutants) [37]. These related diseases include, among others, COPD, ARDS, and asthma. The pathology of COPD is characterized by airway epithelium injury, parenchymal tissue destruction (resulting in emphysema), and disruption of normal repair and defense mechanisms (resulting in small airway fibrosis) [38]. Respiratory epithelium participates in the development of COPD pathogenesis by secreting several pro-inflammatory cytokines and chemokines [39,40], which promote and maintain lung inflammation. Recent studies have demonstrated the importance of EVs for promoting COPD disease by modulating the epithelial-to-mesenchymal transition (EMT) process. Indeed, Xu et al. have shown that CS triggers the modification of the components of bronchial epithelial-derived exosomes and identified exosomal miR-21 derived from bronchial epithelial cells as a mediator of myofibroblast differentiation by targeting the von Hippel–Lindau protein/hypoxia-inducible factor 1α signaling pathway [30]. Downregulation of miR-21 prevented CS-induced airway remodeling [30]. Furthermore, the levels of exosomal miR-21 were high in sera of smokers and COPD patients and are inversely correlated with Forced Expiratory Volume in one second/Forced Vital Capacity (FEV1/FVC), which highlights the potential value of exosomal mir-21 for diagnosis and treatment of COPD. Recently, He et al. have demonstrated that bronchial ECs could generate EVs with less miR-21 when treated with CS extract, alleviating the polarization of M2 macrophages, which indirectly modulate the EMT process in the COPD pathogenesis [41]. Previously, Fujita et al. also observed that CS induced relative upregulation of miR-210 expression in both bronchial ECs and their corresponding EVs [31]. They demonstrated that miR-210 could be transferred into lung fibroblasts via bronchial EC-derived EVs and induce myofibroblast differentiation by silencing the critical autophagy-related factor, ATG7 [31]. This study indicated that CS exposure induced a modification of the composition of bronchial EC-derived EVs and identified exosomal miR-210 as a paracrine autophagy mediator of myofibroblast differentiation. Serban et al. found that CS exposure was sufficient to increase MV levels in plasma of humans and mice, and in supernatants of primary human lung microvascular endothelial cells [17]. CS exposure also altered the composition of endothelial MVs, with an enrichment of let-7d, miR-191, miR-126, and miR-125a [17]. The delivery of those miRNAs to specialized macrophages by endothelial MVs affects the clearance of apoptotic cells. The authors suggested that these targetable effects may be important in the pathogenesis of diseases linked to endothelial injury and inflammation in smokers, such as COPD.

**Figure 2:**
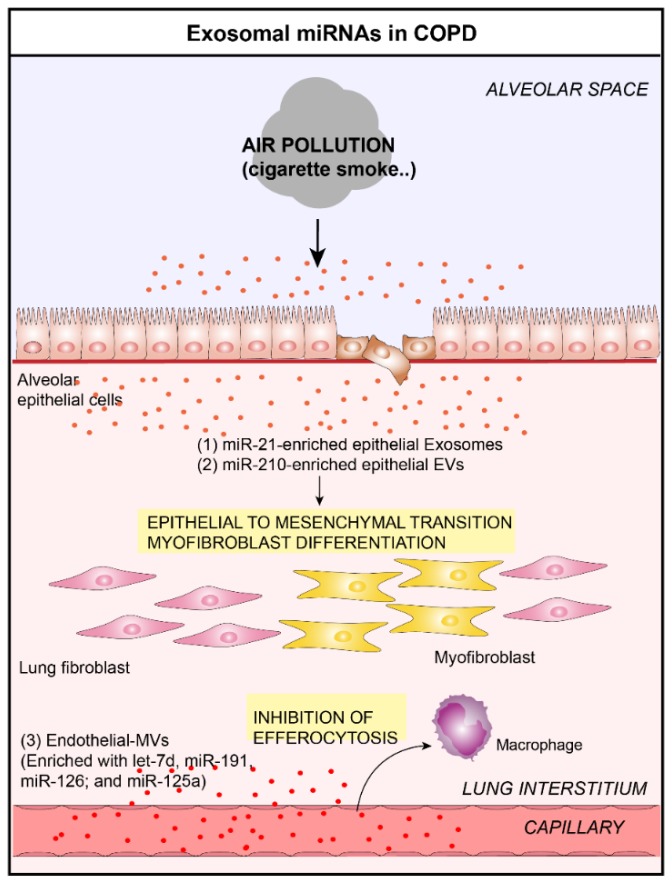
Role of lung EVs in the pathogenesis of Chronic Obstructive Pulmonary Disease (COPD). In the context of COPD, bronchial ECs-derived EVs are able to induce myofibroblast differentiation by the delivery of (**1**) miR-21 [30] and (**2**) miR-210 [31] to lung fibroblasts. (**3**) Furthermore, endothelial-derived EVs are able to inhibit the clearance of apoptotic cells by delivering let-7d, miR-191, miR-126, and miR-125a in lung macrophages [17].

Taken together, all these studies demonstrated that CS exposure induces the alteration of EVs/exosomes composition (e.g., miRNAs), which, in turn, dysregulates several cellular processes involved in the progression of COPD diseases, such as EMT/myofibroblast differentiation (Figure 2).

### 4.2. Asthma and Exosomal microRNAs

**Figure 3:**
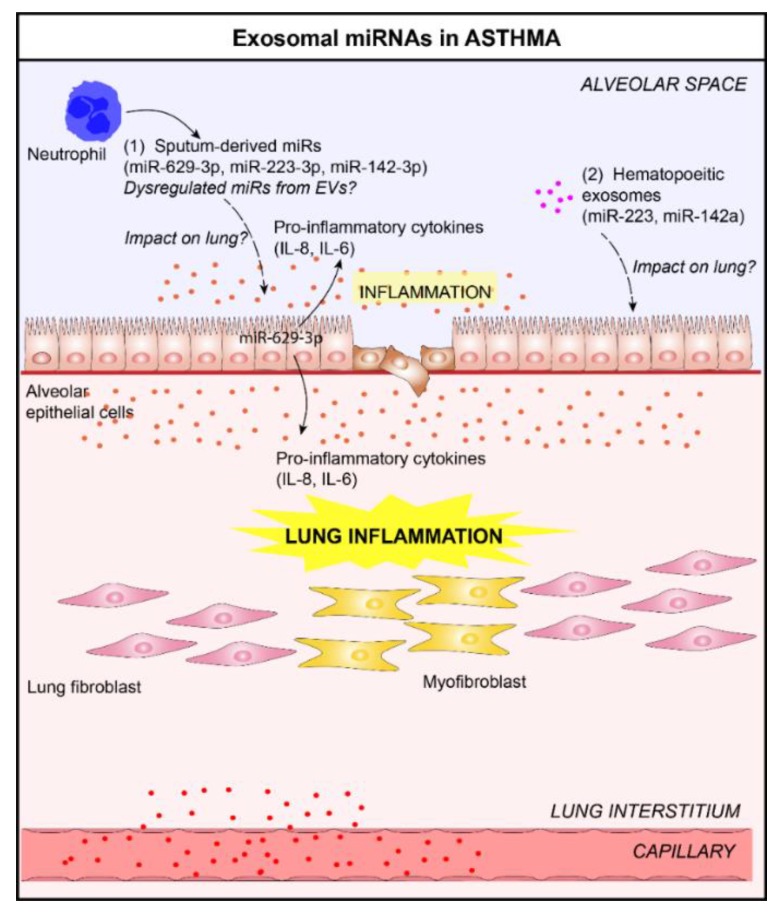
Role of lung EVs in the pathogenesis of asthma. (**1**) In the context of asthma, elevated levels of three sputum microRNAs (miRNAs), miR-629-3p, miR-223-3p, and miR-142-3p, in the sputum of patients with severe asthma, have been reported [47]. The delivery of miR-629-3p in lung ECs could participate in lung inflammation (release of pro-inflammatory cytokines, interleukin (IL)-6 and IL-8, by ECs) [47]. (**2**) The levels of EV-derived miRNAs selectively expressed by immune cells, including miR-223 and miR-142a, increased in bronchoalveolar lavage fluid (BALF) from allergen-treated mice [32]. It would be interesting to study the impact of these exosomal miRNAs on lungs.

Asthma is a chronic inflammatory airway disease characterized by airway hyperresponsiveness and reversible airway obstruction induced by air pollutants and allergens, and increased expression of pro-inflammatory cytokines (such as interleukin (IL)-4, IL-5, and IL-13), which contribute to infiltrating inflammatory cells [42,43]. In asthma, several studies have reported an alteration of miRNA expression in different compartments, including airway biopsies, circulating cells (lymphocytes, neutrophils), and biofluids (peripheral blood, BALF, and sputum supernatants) [44,45,46]. For example, Maes et al. have reported elevated levels of three miRNAs (miR-629-3p, miR-223-3p, and miR-142-3p) in the sputum of patients with severe asthma compared to healthy subjects [47]. The upregulation of miR-629-3p in human bronchial ECs induced the expression of pro-inflammatory cytokine IL-8, suggesting that this miRNA may contribute to neutrophilic asthma inflammatory phenotype. Similarly, the exosomal miRNA content is also altered in asthmatics [24,32,48]. In a recent study, Pua et al. have shown that the composition of extracellular miRNAs of BALF of an untreated mouse was highly correlated with airway-lining epithelium, and found that 80% of detected vesicles were of epithelial origin [32]. After induction of allergic airway inflammation, the levels of EV-derived miRNAs selectively expressed by immune cells, including miR-223 and miR-142a, increased in BALF from allergen-treated mice [32]. They showed that infiltrating immune cells alter the local extracellular environment via the release of EV-derived miRNAs in inflamed tissues. In addition, several miRNAs dysregulated in asthmatic-derived exosomes are associated with inflammation, which suggests that these exosomal miRNAs could impact lung inflammation and contribute to asthma progression (Figure 3).

### 4.3. Acute Lung Injury/Acute Respiratory Distress Syndrome and Exosomal microRNAs

ALI/ARDS is a devastating respiratory disorder, characterized by increased vascular permeability, alveolar hemorrhage, and fibrin deposition [49]. Increasing evidence suggests that macrophages are key factors in the pathogenesis of ALI/ARDS [50]. In ALI/ARDS context, Lee et al. found that acid inhalation in mice induces a remarkable release of epithelium-derived MVs detected in BALF enriched with several miRNAs, including miR-17 and miR-221 [51]. Functional studies revealed that acid-induced epithelial MV-miR-17/221 promotes macrophage β1 integrin recycling and macrophage migration in vitro and recruitment into the lung in vivo, and ultimately contributes to lung inflammation [51]. Several studies have shown that hyperoxia stimulates the release of lung epithelial-derived EVs, which are able to impact the progression of ALI disease [52,53]. For example, Moon et al. have shown that lung epithelial EVs released in hyperoxia condition are able to activate macrophages which promotes neutrophil infiltration in ALI [52]. In another study, Lee et al. showed that hyperoxia up-regulates the levels of certain specific miRNAs in epithelial-derived MVs, including miR-320a and miR-221 [53]. Functionally, the hyperoxia-induced epithelial MVs promote macrophage activation in vitro and facilitate the recruitment of immunomodulatory cells in vivo, which demonstrates that epithelial-derived MVs are able to promote macrophage-regulated lung inflammatory responses via MV-shuttling miRNAs. In a bacterial pneumonia context, macrophages released a rapid and robust amount of ABs enriched in a repertoire of miRNAs, including miR-221 and miR-222 [54]. The delivery of AB-derived miR-221/222 promotes the proliferation of lung ECs by modulating cyclin-dependent kinase inhibitor 1B pathways [54], which could modulate features of lung diseases. In another study, Lee et al. have showed that alveolar EC-derived EVs contribute to innate immune responses after bacterial lung infection [55]. These vesicles actively delivered miRNAs into alveolar macrophages, subsequently promoting inflammasome activation, neutrophil recruitment, and M1-macrophage polarization in response to *P. aeruginosa pneumonia* in vivo.

**Figure 4:**
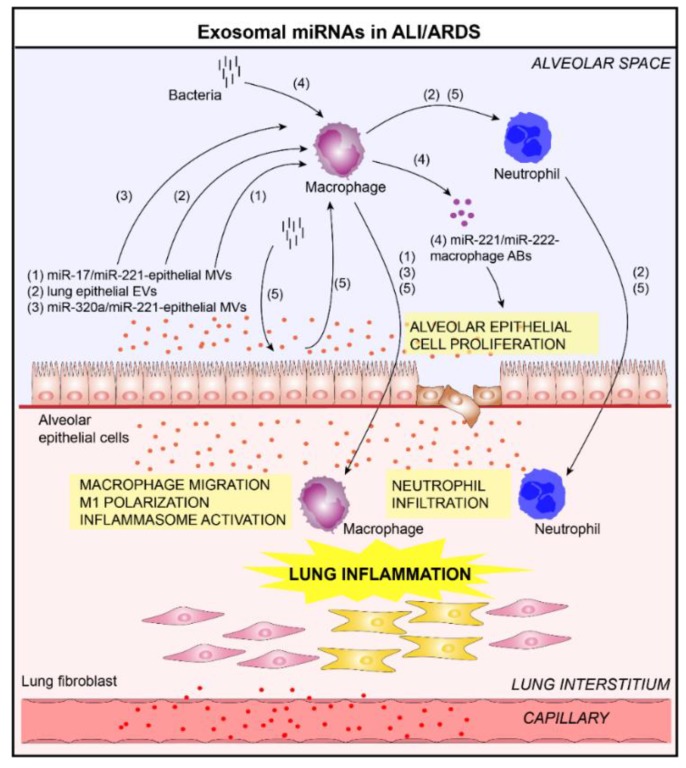
Role of lung EVs in the pathogenesis of Acute Lung Injury/Acute Respiratory Distress Syndrome (ALI/ARDS) (**1–5**). In the context of ALI/ARDS, EVs released by bronchial ECs and alveolar macrophages contribute to lung inflammation. Indeed, these vesicles are able to activate macrophages and promote the infiltration of immune cells (macrophages, neutrophils) in the lung in a miRNA transfer-dependent manner (delivery of (**1**) miR-17/miR-221 [51] and (**3**) miR-320a/miR-221 [53] in alveolar macrophages via epithelial EVs) or (**2**) caspase-3-dependent manner [52]. (**4**) In addition, the alveolar macrophages-derived EVs are able to promote the proliferation of lung ECs by delivering miR-221/222 [54]. (**5**) After bacterial lung infection, alveolar EC-derived EVs contribute to innate immune responses by delivering miRNAs into alveolar macrophages, subsequently promoting inflammasome activation, neutrophil recruitment, and M1-macrophage polarization [55].

In conclusion, EVs/exosomes generated in ALI/ARDS context present pro-inflammatory properties which allow them to activate immune system (e.g., alveolar macrophages) and promote lung inflammation (Figure 4).

### 4.4. Idiopathic Pulmonary Fibrosis and Exosomal microRNAs

**Figure 5:**
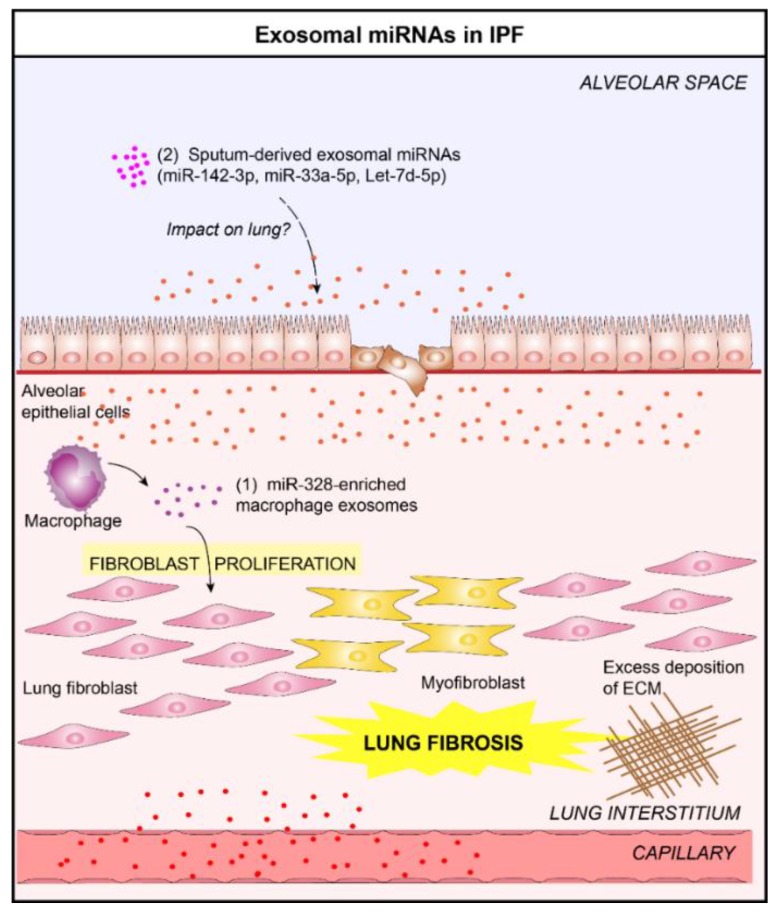
Role of lung EVs in the pathogenesis of Idiopathic Pulmonary Fibrosis (IPF). (**1**) In the context of IPF, macrophage-derived EVs promote the progression of pulmonary fibrosis by delivering miR-328 to pulmonary interstitial fibroblasts which enhances their proliferation [65]. (**2**) Altered levels of three exosomal miRNAs (miR-142-3p, miR-33a-5p, let-7d-5p) in the sputum of IPF patients have been reported [25]. These exosomal miRNAs could play a crucial role in the IPF progression.

IPF is a progressive fibrosing interstitial lung disease of unknown etiology and cure which leads to rapid death within 2–3 years after diagnosis [56,57,58,59,60,61]. IPF is characterized by progressive and irreversible destruction of the lung architecture caused by fibrotic “scar” formation that ultimately leads to organ destruction and death from respiratory failure [62,63]. Its physiopathology remains poorly characterized, although recent studies suggest that this disease results from aberrant dysregulated wound healing response following chronic alveolar epithelial injury and aberrant proliferation of fibroblasts [64]. In the presence of environmental stress, the amount of lung immune cell-derived EVs increases dramatically, with an alteration of their composition (e.g., miRNAs). Several studies have shown that these altered vesicles are responsible for part of the progression of inflammatory- and fibrotic-related lung diseases. In an elegant study, Yao el al. have recently shown that M2 macrophage-derived exosomes overexpressing miR-328 contributed to enhanced pulmonary interstitial fibroblast proliferation and promoted the progression of pulmonary fibrosis through the regulation of FAM13A in a rat model [65]. Previously, we have identified 3 miRNAs showing an aberrant expression in sputum-derived exosomes from IPF patients compared to healthy subjects (miR-142-3p, miR-33a-5p, let-7d-5p) [25]. It could be interesting to study the impact of these miRNAs on IPF progression (Figure 5).

Collectively, these studies reveal that exosomal miRNAs possess a detelerious effect in the context of lung diseases. Their properties (pro-inflammatory or pro-fibrotic) depend on the disease type and its stage, and allow them to promote the progression of lung diseases.

## 5. Exosomes as Promising Diagnostic Biomarkers of Lung Diseases

A growing list of studies have showed that exosomes can be isolated from airway biofluids, including BALF, saliva, and sputum [23,24,25,66]. The remarkable stability of exosomal content in the bloodstream has been attributed to their encapsulation into a bilayer lipid membrane, which protects them from the degrading enzymes (e.g., ribonucleases) present in the biofluids and protects them as a potential biomarker. Accumulating studies have consistently reported the aberrant expression of specific exosomal miRNAs in the blood or airway fluids during the course of lung disease progression, such as COPD, asthma, or IPF (Table 2).

For example, the plasma levels of several skeletal muscle-specific miRNAs (myo-miRNAs) (miR-1, miR-499, miR-133, and miR-206) are elevated in patients with COPD compared to healthy controls [67]. Correlation of altered plasma myo-miRs with skeletal muscle function highlights the potential use of this miRNA signature as biomarker of skeletal muscle dysfunction [67]. In a recent abstract, Burke et al. have reported an alteration of the BALF levels of 23 exosomal miRNAs between COPD and healthy ex-smokers, among those with miR-223-3p, miR-223-5p, miR-338-3p, miR-1469, miR-204-5p, and miR-618 [66]. Interestingly, these differentially expressed exosomal miRNAs are associated to relevant inflammatory pathways (TNFα, NF-κβ, and MAPK signaling) [66], suggesting their impact in the persistent inflammatory response in COPD and therefore are potential targets for future therapies.

As for pulmonary fibrosis disease, we have identified recently 3 miRNAs dysregulated in sputum exosomes from IPF patients compared to healthy subjects (miR-142-3p, miR-33a-5p, let-7d-5p) [25]. Interestingly, we found a negative correlation between miR-142-3p and diffusing capacity of the lungs for carbon monoxide/alveolar volume, suggesting that sputum exosomal miRNAs are associated with the severity of lung fibrosis. This is the first characterization of miRNA content of sputum-derived exosomes in IPF that identified promising biomarkers for diagnosis and disease severity.

In asthma, Maes et al. have also reported elevated levels of miR-142-3p and two other miRNAs, miR-629-3p and miR-223-3p, in sputum of patients with severe asthma compared to healthy subjects [47]. Expression of miR-223-3p and miR-142-3p was associated with airway obstruction (FEV1/FVC). It would be interesting to determine if altered miR-142-3p levels from asthma sputum were from exosomal origins. Levänen et al. described an alteration of exosomal miR profile in BALF from asthmatic patients compared to healthy subjects [24]. Significant differences in BALF exosomal miRNA were detected for 24 miRNAs, including members of the let-7 and miRNA-200 families, providing robust classification of patients with mild asymptomatic asthma from healthy subjects. In another study, elevated levels of miR-128, miR-140-3p, miR-196b-5p, and miR-486-5p were measured in the serum of severe asthmatics in comparison to healthy subjects [48]. Interestingly, the functional analysis of altered miRNAs via *mirPath* software revealed that these miRNAs were associated to ErbB signaling pathway, focal adhesion, and neurotrophin signaling pathway. In addition, several miRNAs associated with immune modulation were identified in exosomes from asthmatic patients, including miR-24, mir-27 [68], and miR-21 [44,45].

However, it is important to note the lack of overlap in the detection of altered exosomal miRNAs. These variations are due in part to the different source biofluids (plasma/serum, BALF, sputum) and technical issues involved in the detection of miRNAs, such as exosome isolation methods (size exclusion chromatography, ultracentrifugation), RNA extraction methods, and miRNA screening methods (small RNA sequencing, RT-qPCR array). Indeed, standardized protocols for isolation, extraction, and quantification of exosomal miRNAs are desperately needed. Recently, the international society of extracellular vesicles (ISEV) has provided guidelines for isolation methods and identification of EVs, including exosomes [9,69], which should contribute on the identification of promising exosomal miRNAs as diagnostic/prognostic biomarkers.

## 6. Exosomes as Promising Therapeutic Tools for Lung Diseases

### 6.1. Mesenchymal Stem Cell-Derived Exosomes Based Therapies

Exosomes derived from mesenchymal stem cells (MSCs) have been proposed as therapeutic tools in many clinical disorders, including lung tissue repair and regeneration after ARDS. Indeed, these vesicles present potential for tissue repair and wound healing [70], and anti-inflammatory and immunosuppressive properties [71,72,73,74].

Several studies have highlighted the immunomodulatory effect of MSC-derived exosomes in asthma. Recently, Du et al. have demonstrated that MSC-derived exosomes are able to upregulate the release of immunosuppressive cytokines IL-10 and TGF-β1 from peripheral blood mononuclear cells, thereby promoting proliferation and immune-suppression capacity of Tregs [75]. In addition, Showalter et al. have established that MSC-derived exosomes are packaged with numerous metabolites that have been directly associated with immunomodulation, including the polarization of M2 macrophages and the induction of regulatory T lymphocytes [76]. MSC-derived EVs also present a protective effect in ALI. Zhu et al. demonstrated that human MSC-derived MVs were therapeutically effective following *E. coli* endotoxin-induced ALI in mice, in part through the expression of Keratinocyte Growth Factor mRNA in the injured alveoli [73]. MVs were able to block inflammation by reducing the influx of neutrophils (73%) and macrophage inflammatory protein-2 levels (49%) in the BALF. In a pig model of influenza virus, Khatri et al. recently demonstrated that MSC-derived EVs possess anti-influenza and anti-inflammatory properties [77]. Indeed, intratracheal administration of MSC-derived EVs 12 h after influenza virus infection significantly reduced virus shedding in the nasal swabs, influenza virus replication in the lungs, and virus-induced production of pro-inflammatory cytokines in the lungs of infected pigs [77]. MSC-derived exosomes from adipose tissue are also able to reduce pathological symptoms in an atopic-dermatitis mouse model and mRNA expression of various inflammatory cytokines (such as IL-4, IL-23, IL-31, and tumor necrosis factor-α) [78].

### 6.2. Exosomes as Natural Drug-Delivery Vehicles

The exploitation of exosomes as drug delivery vehicles offers important advantages compared to other nanoparticulate drug delivery systems, such as liposomes and polymeric nanoparticles [79,80]. Exosomes have a long circulating half-life, the intrinsic ability to target tissues, biocompatibility, and minimal or no inherent toxicity issues [81,82]. Due to their biological properties, exosomes have been proposed as drug- and vaccine-delivery vesicles. For example, plant-derived molecules, such as celastrol and curcumin, are endowed with in vitro antioxidant, immunomodulatory, anti-inflammatory, and anticancer effects, but have a poor solubility. Several groups have investigated the incorporation of these molecules into exosomes, which increases their solubility, their stability, and their bioavailability [83,84,85], and a phase I study is ongoing to investigate the ability of exosomes to deliver curcumin to normal and colon cancer tissue (NCT01294072). In addition, exosomes are efficient biovectors for small RNAs therapies because they are natural vectors of molecular cargoes (e.g., pre-miRNAs, miRNAs) and are able to deliver it to target cells under different pathophysiological contexts [86,87,88]. For example, the study of Ohno et al. was one of the earliest proof-of-concept studies demonstrating that exosomes from human embryonic kidney cells could efficiently deliver exogenous therapeutic let-7a in an EGFR-expressing xenograft breast cancer tissue in RAG2(-/-) mice and induce tumor regression [89].

## 7. Concluding Remarks and Future Perspectives

Interest in the impact of lung exosomes in the modulation of lung homeostasis, as well as their contribution in the progression of a variety of lung diseases, has grown substantially over the past few years. The identification of altered exosomal miRNAs in a lung disease context, as well as the elucidation of their role in the pathogenesis of lung diseases, have provided novel diagnostic biomarkers and therapeutic targets. It is important to mention that the interpretation of biological function of individual altered miRNAs is difficult because miRNAs work together and generally have small individual effects upon gene expression.

Before using these in clinics, several major issues need to be resolved. In order to use exosomal miRNAs as diagnostic/prognostic biomarkers, the protocols for exosome isolation need to be optimized and standardized to minimize the variations due to technical issues. As the effect of miRNAs is fundamentally dependent upon the mRNAs present within the target cell, it is critical to identify the cellular destination of lung exosomes in order to interpret the biological function of exosomal miRNAs. Further investigations need to be performed to identify target cells. Concerning the development of miRNA-based therapies, further investigations need to be performed to improve the delivery of therapeutic miRNAs in specific organs, as well as specific cells, such as alveolar ECs or alveolar macrophages, which play a critical role in the development of lung diseases. We (and others) have begun to explore the use of exosomes loaded with protective cargoes (anti-inflammatory/anti-fibrotic miRNAs) for therapeutic issues.

However, what remains unclear and needs careful investigation is the long-term effect of miRNA mimics/anti-miRNAs since they can persist in the tissues for several months [90] and induce toxicity. Furthermore, one miRNA can target directly several molecular pathways and therefore could induce unintended effects.

Future research will elucidate more deeply the role of exosomes/exosomal miRNAs in the pathogenesis of lung diseases and provide additional information to develop new diagnostics and therapeutics for lung diseases.

## Figures and Tables

**Table 1:** Extracellular vesicle (EV) classification.

Characteristics	Exosomes	MVs	ABs
Size (nm)	30–150	100–1000	1000–5000
Morphology	Cup–shaped	Heterogeneous	Heterogeneous
Density (g/mL)	1.13–1.19	Undetermined	1.16–1.28
Origin	MVBs	Plasma membrane	Plasma membrane
Biogenesis	Fusion of MVBs with plasma membrane	Budding and scission of plasma membrane	Cell fragmentation /blebbing
References	[9,10,11,12]	[9,13,16,17]	[9,14,15]

Abbreviations: ABs, apoptotic bodies; MVs, microvesicles; MVBs, multivesicular bodies.

**Table 2:** EV miRNAs associated with lung diseases.

Lung Diseases	Biofluids	EVs	miRNAs	Expression in Lung Disease (vs. Controls)	References
**COPD**	Plasma	Circulating miRNAs	miR-1 miR-499 miR-133 miR-206	Upregulated Upregulated Upregulated Upregulated	[67]
	BALF	Exosomes	miR-223-3p miR-223-5p miR-338-3p miR-1469 miR-204-5p miR-618	Upregulated Upregulated Upregulated Upregulated Upregulated Upregulated	[66]
	Serum	Exosomes	miR-21	Upregulated	[30]
	Plasma	MVs	let-7d miR-191 miR-126 miR-125a	Upregulated Upregulated Upregulated Upregulated	[17]
**ASTHMA**	Sputum	SputummiRNAs	miR-142-3p miR-629-3p miR-223-3p	Upregulated Upregulated Upregulated	[47]
	BALF	Exosomes	miR-21 miR-1268 miR-658 Let-7a miR-24 miR-26a miR-99a miR-200c	Upregulated Upregulated Downregulated Downregulated Downregulated Downregulated Downregulated Downregulated	[24]
	Serum	Exosomes	miR-128 miR-140-3p miR-196-5p miR-468-5p	Upregulated Upregulated Upregulated Upregulated	[48]
**IPF**	Sputum	Exosomes	miR-142-3p miR-33a-5p Let-7d-5p	Upregulated Upregulated Downregulated	[25]

Abbreviations: BALF, bronchoalveolar lavage fluid; COPD, chronic obstructive pulmonary disease; EVs, extracellular vesicles; IPF, idiopathic pulmonary fibrosis; miRNAs, microRNAs; MVs, microvesicles.

## Abbreviations

ABs	apoptotic bodies
BALF	bronchoalveolar lavage fluid
CS	cigarette smokeECs: epithelial cells
EMT	epithelial-to-mesenchymal transition
FIRS	forum of international respiratory societies
IPF	idiopathic pulmonary fibrosis
miRNA	micro
MUC	mucin
MVBs	multivesicular bodies
ALI/ARDS	acute lung injury/acute respiratory distress syndrome
COPD	chronic obstructive pulmonary disease
EVs	extracellular vesicles
IL	interleukin
ISEV	international society of extracellular vesicles
RNAMSCs	mesenchymal stem cells
MVs	microvesicles
SOCS	suppressor of cytokine signaling

## References

[1] European Respiratory Society (2017). Forum of International Respiratory Societies.

[2] (2012). WHO | Global Surveillance, Prevention and Control of Chronic Respiratory Diseases: A Comprehensive Approach.

[3] Burney P.G.J., Patel J., Newson R., Minelli C., Naghavi M. (2015). Global and regional trends in COPD mortality, 1990–2010. Eur. Respir. J..

[4] Global Asthma Report 2014. http://www.globalasthmanetwork.org/news/GAR2014.php.

[5] Wardlaw T.M., Johansson E.W., Hodge M.J., UNICEF, Division of Communication, World Health Organization (2006). Pneumonia: The Forgotten Killer of Children.

[6] Tata P.R., Rajagopal J. (2017). Plasticity in the lung: Making and breaking cell identity. Development.

[7] Shaykhiev R., Otaki F., Bonsu P., Dang D.T., Teater M., Strulovici-Barel Y., Salit J., Harvey B.-G., Crystal R.G. (2011). Cigarette smoking reprograms apical junctional complex molecular architecture in the human airway epithelium in vivo. Cell. Mol. Life Sci..

[8] Guiot J., Bondue B., Henket M., Corhay J.L., Louis R. (2016). Raised serum levels of IGFBP-1 and IGFBP-2 in idiopathic pulmonary fibrosis. BMC Pulm. Med..

[9] Théry C., Witwer K.W., Aikawa E., Alcaraz M.J., Anderson J.D., Andriantsitohaina R., Antoniou A., Arab T., Archer F., Atkin-Smith G.K. (2018). Minimal information for studies of extracellular vesicles 2018 (MISEV2018): A position statement of the International Society for Extracellular Vesicles and update of the MISEV2014 guidelines. J. Extracell. Vesicles.

[10] Kowal J., Tkach M., Théry C. (2014). Biogenesis and secretion of exosomes. Curr. Opin. Cell Biol..

[11] Henne W.M., Stenmark H., Emr S.D. (2013). Molecular mechanisms of the membrane sculpting ESCRT pathway. Cold Spring Harb. Perspect. Biol..

[12] Trajkovic K., Hsu C., Chiantia S., Rajendran L., Wenzel D., Wieland F., Schwille P., Brügger B., Simons M. (2008). Ceramide Triggers Budding of Exosome Vesicles into Multivesicular Endosomes. Science.

[13] Simoncini S., Njock M.-S., Robert S., Camoin-Jau L., Sampol J., Harlé J.-R., Nguyen C., Dignat-George F., Anfosso F. (2009). TRAIL/Apo2L Mediates the Release of Procoagulant Endothelial Microparticles Induced by Thrombin In Vitro. Circ. Res..

[14] Kerr J.F., Wyllie A.H., Currie A.R. (1972). Apoptosis: A basic biological phenomenon with wide-ranging implications in tissue kinetics. Br. J. Cancer.

[15] Atkin-Smith G.K., Tixeira R., Paone S., Mathivanan S., Collins C., Liem M., Goodall K.J., Ravichandran K.S., Hulett M.D., Poon I.K.H. (2015). A novel mechanism of generating extracellular vesicles during apoptosis via a beads-on-a-string membrane structure. Nat. Commun..

[16] Leroyer A.S., Anfosso F., Lacroix R., Sabatier F., Simoncini S., Njock S.M., Jourde N., Brunet P., Camoin-Jau L., Sampol J. (2010). Endothelial-derived microparticles: Biological conveyors at the crossroad of inflammation, thrombosis and angiogenesis. Thromb. Haemost..

[17] Serban K.A., Rezania S., Petrusca D.N., Poirier C., Cao D., Justice M.J., Patel M., Tsvetkova I., Kamocki K., Mikosz A. (2016). Structural and functional characterization of endothelial microparticles released by cigarette smoke. Sci. Rep..

[18] Harding C., Heuser J., Stahl P. (1983). Receptor-mediated endocytosis of transferrin and recycling of the transferrin receptor in rat reticulocytes. J. Cell Biol..

[19] Pan B.T., Teng K., Wu C., Adam M., Johnstone R.M. (1985). Electron microscopic evidence for externalization of the transferrin receptor in vesicular form in sheep reticulocytes. J. Cell Biol..

[20] Valadi H., Ekström K., Bossios A., Sjöstrand M., Lee J.J., Lötvall J.O. (2007). Exosome-mediated transfer of mRNAs and microRNAs is a novel mechanism of genetic exchange between cells. Nat. Cell Biol..

[21] Andreu Z., Yáñez-Mó M. (2014). Tetraspanins in extracellular vesicle formation and function. Front. Immunol..

[22] Colombo M., Moita C., van Niel G., Kowal J., Vigneron J., Benaroch P., Manel N., Moita L.F., Théry C., Raposo G. (2013). Analysis of ESCRT functions in exosome biogenesis, composition and secretion highlights the heterogeneity of extracellular vesicles. J. Cell Sci..

[23] Admyre C., Grunewald J., Thyberg J., Gripenbäck S., Tornling G., Eklund A., Scheynius A., Gabrielsson S. (2003). Exosomes with major histocompatibility complex class II and co-stimulatory molecules are present in human BAL fluid. Eur. Respir. J..

[24] Levänen B., Bhakta N.R., Torregrosa Paredes P., Barbeau R., Hiltbrunner S., Pollack J.L., Sköld C.M., Svartengren M., Grunewald J., Gabrielsson S. (2013). Altered microRNA profiles in bronchoalveolar lavage fluid exosomes in asthmatic patients. J. Allergy Clin. Immunol..

[25] Njock M.-S., Guiot J., Henket M.A., Nivelles O., Thiry M., Dequiedt F., Corhay J.-L., Louis R.E., Struman I. (2019). Sputum exosomes: Promising biomarkers for idiopathic pulmonary fibrosis. Thorax.

[26] Gallo A., Tandon M., Alevizos I., Illei G.G. (2012). The majority of microRNAs detectable in serum and saliva is concentrated in exosomes. PLoS ONE.

[27] Guiot J., Demarche S., Henket M., Paulus V., Graff S., Schleich F., Corhay J.-L., Louis R., Moermans C. (2017). Methodology for Sputum Induction and Laboratory Processing. J. Vis. Exp..

[28] Njock M.-S., Cheng H.S., Dang L.T., Nazari-Jahantigh M., Lau A.C., Boudreau E., Roufaiel M., Cybulsky M.I., Schober A., Fish J.E. (2015). Endothelial cells suppress monocyte activation through secretion of extracellular vesicles containing antiinflammatory microRNAs. Blood.

[29] Bovy N., Blomme B., Frères P., Dederen S., Nivelles O., Lion M., Carnet O., Martial J.A., Noël A., Thiry M. (2015). Endothelial exosomes contribute to the antitumor response during breast cancer neoadjuvant chemotherapy via microRNA transfer. Oncotarget.

[30] Xu H., Ling M., Xue J., Dai X., Sun Q., Chen C., Liu Y., Zhou L., Liu J., Luo F. (2018). Exosomal microRNA-21 derived from bronchial epithelial cells is involved in aberrant epithelium-fibroblast cross-talk in COPD induced by cigarette smoking. Theranostics.

[31] Fujita Y., Araya J., Ito S., Kobayashi K., Kosaka N., Yoshioka Y., Kadota T., Hara H., Kuwano K., Ochiya T. (2015). Suppression of autophagy by extracellular vesicles promotes myofibroblast differentiation in COPD pathogenesis. J. Extracell. Vesicles.

[32] Pua H.H., Happ H.C., Gray C.J., Mar D.J., Chiou N.-T., Hesse L.E., Ansel K.M. (2019). Increased Hematopoietic Extracellular RNAs and Vesicles in the Lung during Allergic Airway Responses. Cell Rep..

[33] Kesimer M., Scull M., Brighton B., DeMaria G., Burns K., O’Neal W., Pickles R.J., Sheehan J.K. (2009). Characterization of exosome-like vesicles released from human tracheobronchial ciliated epithelium: A possible role in innate defense. FASEB J..

[34] Gupta R., Radicioni G., Abdelwahab S., Dang H., Carpenter J., Chua M., Mieczkowski P.A., Sheridan J.T., Randell S.H., Kesimer M. (2019). Intercellular Communication between Airway Epithelial Cells Is Mediated by Exosome-Like Vesicles. Am. J. Respir. Cell Mol. Biol..

[35] Bourdonnay E., Zasłona Z., Penke L.R.K., Speth J.M., Schneider D.J., Przybranowski S., Swanson J.A., Mancuso P., Freeman C.M., Curtis J.L. (2015). Transcellular delivery of vesicular SOCS proteins from macrophages to epithelial cells blunts inflammatory signaling. J. Exp. Med..

[36] Ismail N., Wang Y., Dakhlallah D., Moldovan L., Agarwal K., Batte K., Shah P., Wisler J., Eubank T.D., Tridandapani S. (2013). Macrophage microvesicles induce macrophage differentiation and miR-223 transfer. Blood.

[37] Barratt S., Creamer A., Hayton C., Chaudhuri N. (2018). Idiopathic Pulmonary Fibrosis (IPF): An Overview. J. Clin. Med..

[38] Vestbo J., Hurd S.S., Agustí A.G., Jones P.W., Vogelmeier C., Anzueto A., Barnes P.J., Fabbri L.M., Martinez F.J., Nishimura M. (2013). Global Strategy for the Diagnosis, Management, and Prevention of Chronic Obstructive Pulmonary Disease. Am. J. Respir. Crit. Care Med..

[39] Chuquimia O.D., Petursdottir D.H., Rahman M.J., Hartl K., Singh M., Fernández C. (2012). The role of alveolar epithelial cells in initiating and shaping pulmonary immune responses: Communication between innate and adaptive immune systems. PLoS ONE.

[40] Witherden I.R., Vanden Bon E.J., Goldstraw P., Ratcliffe C., Pastorino U., Tetley T.D. (2004). Primary Human Alveolar Type II Epithelial Cell Chemokine Release. Am. J. Respir. Cell Mol. Biol..

[41] He S., Chen D., Hu M., Zhang L., Liu C., Traini D., Grau G.E., Zeng Z., Lu J., Zhou G. (2019). Bronchial epithelial cell extracellular vesicles ameliorate epithelial–mesenchymal transition in COPD pathogenesis by alleviating M2 macrophage polarization. Nanomed. Nanotechnol. Biol. Med..

[42] Wenzel S.E. (2012). Asthma phenotypes: The evolution from clinical to molecular approaches. Nat. Med..

[43] Lambrecht B.N., Hammad H. (2015). The immunology of asthma. Nat. Immunol..

[44] Lu T.X., Munitz A., Rothenberg M.E. (2009). MicroRNA-21 is up-regulated in allergic airway inflammation and regulates IL-12p35 expression. J. Immunol..

[45] Lu T.X., Hartner J., Lim E.-J., Fabry V., Mingler M.K., Cole E.T., Orkin S.H., Aronow B.J., Rothenberg M.E. (2011). MicroRNA-21 Limits In Vivo Immune Response-Mediated Activation of the IL-12/IFN-γ Pathway, Th1 Polarization, and the Severity of Delayed-Type Hypersensitivity. J. Immunol..

[46] Wu X.-B., Wang M.-Y., Zhu H.-Y., Tang S.-Q., You Y.-D., Xie Y.-Q. (2014). Overexpression of microRNA-21 and microRNA-126 in the patients of bronchial asthma. Int. J. Clin. Exp. Med..

[47] Maes T., Cobos F.A., Schleich F., Sorbello V., Henket M., De Preter K., Bracke K.R., Conickx G., Mesnil C., Vandesompele J. (2016). Asthma inflammatory phenotypes show differential microRNA expression in sputum. J. Allergy Clin. Immunol..

[48] Suzuki M., Konno S., Makita H., Shimizu K., Kimura H., Kimura H., Nishimura M. (2016). Altered circulating exosomal RNA profiles detected by next-generation sequencing in patients with severe asthma. Proceedings of the 3.1 Molecular Pathology and Functional Genomics.

[49] Matuschak G.M., Lechner A.J. (2010). Acute lung injury and the acute respiratory distress syndrome: Pathophysiology and treatment. Mo. Med..

[50] Johnston L.K., Rims C.R., Gill S.E., McGuire J.K., Manicone A.M. (2012). Pulmonary macrophage subpopulations in the induction and resolution of acute lung injury. Am. J. Respir. Cell Mol. Biol..

[51] Lee H., Zhang D., Wu J., Otterbein L.E., Jin Y. (2017). Lung Epithelial Cell–Derived Microvesicles Regulate Macrophage Migration via MicroRNA-17/221–Induced Integrin β _1_ Recycling. J. Immunol..

[52] Moon H.-G., Cao Y., Yang J., Lee J.H., Choi H.S., Jin Y. (2015). Lung epithelial cell-derived extracellular vesicles activate macrophage-mediated inflammatory responses via ROCK1 pathway. Cell Death Dis..

[53] Lee H., Zhang D., Zhu Z., Dela Cruz C.S., Jin Y. (2016). Epithelial cell-derived microvesicles activate macrophages and promote inflammation via microvesicle-containing microRNAs. Sci. Rep..

[54] Zhu Z., Zhang D., Lee H., Menon A.A., Wu J., Hu K., Jin Y. (2017). Macrophage-derived apoptotic bodies promote the proliferation of the recipient cells via shuttling microRNA-221/222. J. Leukoc. Biol..

[55] Lee H., Groot M., Pinilla-Vera M., Fredenburgh L.E., Jin Y. (2019). Identification of miRNA-rich vesicles in bronchoalveolar lavage fluid: Insights into the function and heterogeneity of extracellular vesicles. J. Control. Release Off. J. Control. Release Soc..

[56] Raghu G., Collard H.R., Egan J.J., Martinez F.J., Behr J., Brown K.K., Colby T.V., Cordier J.-F., Flaherty K.R., Lasky J.A. (2011). An Official ATS/ERS/JRS/ALAT Statement: Idiopathic Pulmonary Fibrosis: Evidence-based Guidelines for Diagnosis and Management. Am. J. Respir. Crit. Care Med..

[57] Noble P.W., Albera C., Bradford W.Z., Costabel U., Glassberg M.K., Kardatzke D., King T.E., Lancaster L., Sahn S.A., Szwarcberg J. (2011). Pirfenidone in patients with idiopathic pulmonary fibrosis (CAPACITY): Two randomised trials. Lancet.

[58] Guiot J., Henket M., Corhay J.L., Moermans C., Louis R. (2017). Sputum biomarkers in IPF: Evidence for raised gene expression and protein level of IGFBP-2, IL-8 and MMP-7. PLoS ONE.

[59] King T.E., Pardo A., Selman M. (2011). Idiopathic pulmonary fibrosis. Lancet.

[60] Guiot J., Duysinx B., Seidel L., Henket M., Gester F., Bonhomme O., Corhay J.-L., Louis R. (2018). Clinical experience in idiopathic pulmonary fibrosis: A retrospective study. Acta Clin. Belg..

[61] Guiot J., Moermans C., Henket M., Corhay J.-L., Louis R. (2017). Blood Biomarkers in Idiopathic Pulmonary Fibrosis. Lung.

[62] Wynn T.A. (2011). Integrating mechanisms of pulmonary fibrosis. J. Exp. Med..

[63] Lederer D.J., Martinez F.J. (2018). Idiopathic Pulmonary Fibrosis. New Engl. J. Med..

[64] Wilson M.S., Wynn T.A. (2009). Pulmonary fibrosis: Pathogenesis, etiology and regulation. Mucosal Immunol..

[65] Yao M.-Y., Zhang W.-H., Ma W.-T., Liu Q.-H., Xing L.-H., Zhao G.-F. (2019). microRNA-328 in exosomes derived from M2 macrophages exerts a promotive effect on the progression of pulmonary fibrosis via FAM13A in a rat model. Exp. Mol. Med..

[66] Burke H., Heinson A., Freeman A., Ostridge K., Watson A., Staples K., Spalluto M., Wilkinson T. (2018). Late Breaking Abstract–Differentially expressed exosomal miRNAs target key inflammatory pathways in COPD. Proceedings of the Airway Cell Biology and Immunopathology.

[67] Donaldson A., Natanek S.A., Lewis A., Man W.D.-C., Hopkinson N.S., Polkey M.I., Kemp P.R. (2013). Increased skeletal muscle-specific microRNA in the blood of patients with COPD. Thorax.

[68] Pua H.H., Steiner D.F., Patel S., Gonzalez J.R., Ortiz-Carpena J.F., Kageyama R., Chiou N.-T., Gallman A., de Kouchkovsky D., Jeker L.T. (2016). MicroRNAs 24 and 27 Suppress Allergic Inflammation and Target a Network of Regulators of T Helper 2 Cell-Associated Cytokine Production. Immunity.

[69] Witwer K.W., Buzás E.I., Bemis L.T., Bora A., Lässer C., Lötvall J., Nolte-‘t Hoen E.N., Piper M.G., Sivaraman S., Skog J. (2013). Standardization of sample collection, isolation and analysis methods in extracellular vesicle research. J. Extracell. Vesicles.

[70] Matthay M.A. (2015). Therapeutic Potential of Mesenchymal Stromal Cells for Acute Respiratory Distress Syndrome. Ann. Am. Thorac. Soc..

[71] Griffin M.D., Ryan A.E., Alagesan S., Lohan P., Treacy O., Ritter T. (2013). Anti-donor immune responses elicited by allogeneic mesenchymal stem cells: What have we learned so far?. Immunol. Cell Biol..

[72] Monsel A., Zhu Y., Gennai S., Hao Q., Hu S., Rouby J.-J., Rosenzwajg M., Matthay M.A., Lee J.W. (2015). Therapeutic Effects of Human Mesenchymal Stem Cell–derived Microvesicles in Severe Pneumonia in Mice. Am. J. Respir. Crit. Care Med..

[73] Zhu Y.-G., Feng X.-M., Abbott J., Fang X.-H., Hao Q., Monsel A., Qu J.-M., Matthay M.A., Lee J.W. (2014). Human mesenchymal stem cell microvesicles for treatment of Escherichia coli endotoxin-induced acute lung injury in mice. Stem Cells.

[74] Lai P., Chen X., Guo L., Wang Y., Liu X., Liu Y., Zhou T., Huang T., Geng S., Luo C. (2018). A potent immunomodulatory role of exosomes derived from mesenchymal stromal cells in preventing cGVHD. J. Hematol. Oncol..

[75] Du Y.-M., Zhuansun Y.-X., Chen R., Lin L., Lin Y., Li J.-G. (2018). Mesenchymal stem cell exosomes promote immunosuppression of regulatory T cells in asthma. Exp. Cell Res..

[76] Showalter M.R., Wancewicz B., Fiehn O., Archard J.A., Clayton S., Wagner J., Deng P., Halmai J., Fink K.D., Bauer G. (2019). Primed mesenchymal stem cells package exosomes with metabolites associated with immunomodulation. Biochem. Biophys. Res. Commun..

[77] Khatri M., Richardson L.A., Meulia T. (2018). Mesenchymal stem cell-derived extracellular vesicles attenuate influenza virus-induced acute lung injury in a pig model. Stem Cell Res. Ther..

[78] Cho B.S., Kim J.O., Ha D.H., Yi Y.W. (2018). Exosomes derived from human adipose tissue-derived mesenchymal stem cells alleviate atopic dermatitis. Stem Cell Res. Ther..

[79] Sercombe L., Veerati T., Moheimani F., Wu S.Y., Sood A.K., Hua S. (2015). Advances and Challenges of Liposome Assisted Drug Delivery. Front. Pharmacol..

[80] Agrawal U., Sharma R., Gupta M., Vyas S.P. (2014). Is nanotechnology a boon for oral drug delivery?. Drug Discov. Today.

[81] Raemdonck K., Braeckmans K., Demeester J., De Smedt S.C. (2014). Merging the best of both worlds: Hybrid lipid-enveloped matrix nanocomposites in drug delivery. Chem. Soc. Rev..

[82] Ha D., Yang N., Nadithe V. (2016). Exosomes as therapeutic drug carriers and delivery vehicles across biological membranes: Current perspectives and future challenges. Acta Pharm. Sinica. B.

[83] Aqil F., Munagala R., Jeyabalan J., Agrawal A.K., Gupta R. (2017). Exosomes for the Enhanced Tissue Bioavailability and Efficacy of Curcumin. Aaps J..

[84] Vashisht M., Rani P., Onteru S.K., Singh D. (2017). Curcumin Encapsulated in Milk Exosomes Resists Human Digestion and Possesses Enhanced Intestinal Permeability in Vitro. Appl. Biochem. Biotechnol..

[85] Aqil F., Kausar H., Agrawal A.K., Jeyabalan J., Kyakulaga A.-H., Munagala R., Gupta R. (2016). Exosomal formulation enhances therapeutic response of celastrol against lung cancer. Exp. Mol. Pathol..

[86] Melo S.A., Sugimoto H., O’Connell J.T., Kato N., Villanueva A., Vidal A., Qiu L., Vitkin E., Perelman L.T., Melo C.A. (2014). Cancer Exosomes Perform Cell-Independent MicroRNA Biogenesis and Promote Tumorigenesis. Cancer Cell.

[87] Zhou W., Fong M.Y., Min Y., Somlo G., Liu L., Palomares M.R., Yu Y., Chow A., O’Connor S.T.F., Chin A.R. (2014). Cancer-Secreted miR-105 Destroys Vascular Endothelial Barriers to Promote Metastasis. Cancer Cell.

[88] Aga M., Bentz G.L., Raffa S., Torrisi M.R., Kondo S., Wakisaka N., Yoshizaki T., Pagano J.S., Shackelford J. (2014). Exosomal HIF1α supports invasive potential of nasopharyngeal carcinoma-associated LMP1-positive exosomes. Oncogene.

[89] Ohno S., Takanashi M., Sudo K., Ueda S., Ishikawa A., Matsuyama N., Fujita K., Mizutani T., Ohgi T., Ochiya T. (2013). Systemically injected exosomes targeted to EGFR deliver antitumor microRNA to breast cancer cells. Mol. Ther. J. Am. Soc. Gene Ther..

[90] Elmén J., Lindow M., Schütz S., Lawrence M., Petri A., Obad S., Lindholm M., Hedtjärn M., Hansen H.F., Berger U. (2008). LNA-mediated microRNA silencing in non-human primates. Nature.

